# Prognostic Impact of Programmed Death-Ligand 1 Determination in Unresectable Locally Advanced Head and Neck Squamous Cell Carcinoma: A Retrospective Analysis in a Portuguese Centre

**DOI:** 10.7759/cureus.79084

**Published:** 2025-02-16

**Authors:** Luís Guilherme Santos, Ana Rita Garcia, Margarida Teixeira

**Affiliations:** 1 Medical Oncology Department, Instituto Português de Oncologia de Coimbra Francisco Gentil, Coimbra, PRT

**Keywords:** head and neck oncology, head and neck squamous cell carcinoma (hnscc), immuno-checkpoint inhibitor, programmed death ligand-1, recurrence-free survival

## Abstract

Locally advanced head and neck squamous cell carcinoma (HNSCC) is treated with definitive concurrent chemoradiation if deemed unresectable. Contrarily to the metastatic/recurrent setting, there is no current role for immunotherapy in the locally advanced setting, all trials being negative in their primary endpoints. As such, and although it may still be performed, determination of programmed death-ligand 1 (PD-L1) is not mandatory in locally advanced disease. We aimed to assess if there was any correlation with PD-L1 positivity (when obtained), disease characteristics, and recurrence-free survival in unresectable, locally advanced HNSCC eligible for concurrent chemoradiation in a Portuguese centre. We retrospectively analysed 164 patients for five years, most of whom had unresectable stage IV disease treated with cisplatin-based chemoradiation. PD-L1 was determined in 35% of patients. While it did not correlate to anatomical disease location, treatment tolerance, p16 status, or clinical staging at diagnosis, PD-L1 over-expression seemed to identify a group of patients in which recurrence-free survival was shorter, highlighting the need for continued clinical trials assessing the role of PD-L1 testing and immunotherapy in this setting.

## Introduction

Head and neck squamous cell carcinoma (HNSCC) is considered the seventh most prevalent cancer worldwide with approximately 890,000 cases a year, representing approximately 4.5% of cancer diagnoses and deaths [1]. Tobacco and alcohol consumption are the main risk factors associated with HNSCC and have a synergistic effect [2]. Infection by human papillomavirus (HPV) has been associated with an increase in risk, especially in SCC of the oropharynx, and HPV-related HNSCC is increasing in incidence, particularly in the developed world [3]. HNSCC is frequently an aggressive disease requiring intense treatment regimens. An estimated 60% of cases [4] are considered locally advanced (LA) at diagnosis and treatment according to current guidelines may consist of either surgery, whenever resectable, followed by adjuvant chemoradiation (CRT) if major recurrence risk factors are present (namely extracapsular extension and/or positive margins) or concomitant definitive CRT if the disease is unresectable [5].

Over-expression of programmed death-ligand 1 (PD-L1) in cancer cells inhibits the cytotoxic effect of immune cells leading to immune system evasion, unchecked proliferation, and tumour growth. Immune checkpoint inhibitors (ICIs) are a staple of treatment for PD-L1 over-expressed disease and revolutionized prognosis across a great variety of cancers in every setting. In HNSCC, current guidelines established ICIs as a standard of care first-line treatment in PD-L1 positive patients in recurrent/metastatic settings based on the KEYNOTE-040 and 048 trials [6,7], either combined with chemotherapy or in monotherapy. Determination of PD-L1 through immunohistochemistry (IHC) and calculation of combined positive score (CPS) is, therefore, mandatory in this setting. 

The success of immunotherapy in the recurrent/metastatic setting led to trials exploring ICI in the LA setting. While some retrospective data pointed to the benefit of immunotherapy on PD-L1 positive patients in this setting, results from the phase III JAVELIN Head and Neck 100 [8], KEYNOTE-412 [9], and GORTEC-REACH [10] trials have been disappointing, and their endpoints were negative. As such, state-of-the-art treatment in LA HNSCC has not changed. 

Although not mandatory in the LA setting, determination of PD-L1 overexpression and CPS score might still be performed depending on institutional protocols and indeed is frequently performed in our centre. While it has no clinical relevance in determining the choice of treatment, PD-L1 analyses at this stage might be useful to save time on electing treatment once, and if, there is disease recurrence or progression.

Our team sought to find out if patients diagnosed with unresectable, LA HNSCC at a Portuguese Oncology Centre, had information regarding PD-L1 and CPS status at the moment of diagnosis, and if so, how did this correlate to the characteristics of the disease and the patient, and if there was any impact in prognosis, through the analysis of recurrence-free survival (RFS). Furthermore, we sought to answer the question of the true utility of requesting this analysis, considering the considerable associated cost and lack of impact in initial treatment besides a time-saving strategy.

## Materials and methods

This was a retrospective study conducted at the Instituto Português de Oncologia de Coimbra Francisco Gentil, Coimbra, Portugal between January 2019 and June 2024. A total of 164 patients were included in the study.

Eligibility criteria

Patients diagnosed with unresectable LA HNSCC who were eligible for definitive chemoradiation treatment during the study period were included. Patients with a diagnosis of SCC of the nasopharynx, the upper third of the oesophagus, and other histological variants or locations were excluded, as were any patients included in any clinical trials at the time of diagnosis.

Data collection

Data were collected from the medical records. Median follow-up was 10.4 months, with a minimum of six months. Each assessed patient was discussed previous to the start of treatment at a multidisciplinary reunion in which unresectability of disease was confirmed and contraindications to cisplatin-based chemotherapy were excluded. Assessed variables in each patient were sex, age at the time of diagnosis, history of tobacco and alcohol consumption, anatomical location of the disease (oropharynx, hypopharynx, larynx, oral cavity or unknown primary), clinical staging according to the American Joint Committee on Cancer (AJCC), 8th Edition, p16 status [5] in the biopsy specimen, PD-L1 status trough IHC analysis and CPS score whenever available, proposed cisplatin-based treatment and the number of treatment cycles completed, main toxicities attributable to treatment and toxicity grading, and time to recurrence/progression after the end of the last treatment cycle.

Statistical analysis

All data was analysed using IBM SPSS Statistics for Windows, Version 19.0 (2024; IBM Corp., Armonk, New York, United States), using contingency tables and chi-square tests for a significance level of 0.05. RFS was studied through Kaplan-Meier curves for a level of significance of 0.05.

## Results

The sociodemographic and pathological characteristics of the patients are shown in Table 1. The majority were male (n=155, 95%), and the median age was 59 years (range, 38-78 years). A total of 122 patients (74%) were described as smokers while 115 (70%) had a history of alcohol consumption. SCC of the oropharynx constituted the majority of patients (n=60, 37%), of which 17 (10%) were p16 positive. SCC of the hypopharynx was the second most common diagnosis (n=41, 25%), followed by SCC of the oral cavity (n=38, 23%), larynx (n=21, 13%), and occult primary (n=4, 2%). Most patients (n=138, 84%) had clinical stage IV disease (93 patients (57%) stage IVa and 45 patients (27%) stage IVb), while 22 (13%) patients had clinical stage III disease, and only four (2%) were clinical stage II. 

**Table 1:** Sociodemographic and pathological characteristics of participants (N = 164) SCC: squamous cell carcinoma; AJCC: American Joint Committee on Cancer; PD-L1: programmed death-ligand 1; CPS: combined positive score; CTCAE: Common Terminology Criteria for Adverse Events; RFS: recurrence-free survival

Characteristics	Values
Age (years), median (SD; interval)	59 (8; 38-78)
Sex, n (%)	
Male	155 (95)
Female	9 (5)
Smoking habit, n (%)	
Yes	122 (74)
No	42 (26)
Alcohol consumption, n (%)	
Yes	115 (70)
No	49 (30)
Diagnosis according to anatomical location, n (%)	
SCC oropharynx	60 (37)
SCC hypopharynx	41 (25)
SCC oral cavity	38 (23)
SCC larynx	21 (13)
SCC occult primary	4 (2)
Staging (AJCC 8^th^ Edition), n (%)	
II	4 (2)
III	22 (13)
IVa	93 (57)
IVb	45 (27)
p16 status, n (%)	
Positive	22 (13)
Negative/non-determined	142 (87)
PD-L1 assessment performed, n (%)	
Yes	62 (38)
No	102 (62)
PD-L1 status, n (%)	
Non-determined	102 (62)
Determined: positive	57 (35)
Determined: negative	5 (3)
CPS Interval, n (%)	
Negative/non-determined	107 (65)
1-19	30 (18)
≥19	27 (17)
Cisplatin cycles completed, n (%)	
One	43 (26)
Two	82 (50)
Three	39 (24)
Radiotherapy treatment completed, n (%)	
Yes	162 (99)
No	2 (1)
Graduation of toxicity according to CTCAE, n (%)	
0/no relevant toxicity	41 (25)
1	2 (1)
2	77 (47)
3	39 (24)
4	5 (3)
Disease recurrence, n (%)	
Yes	95 (58)
No	69 (42)
RFS, median (SD; interval)	18 (17; 1-59)

All patients were offered CRT after a multidisciplinary discussion in which unresectability was confirmed and absolute contraindications for platin-based treatment were excluded. The proposed treatment regimen in all cases consisted of standard-of-care cisplatin 100 mg/m^2^ given intravenously on days 1, 22, and 43, concomitant with radiotherapy (total cumulative dose of 70 Gray). 

A total of 122 patients (74%) had clinically relevant treatment-related toxicities mainly attributed to chemotherapy and requiring postponement or suspension of treatment cycles (median of completed cisplatin cycles: 2). Non-febrile neutropenia was the most common toxicity (39 patients, approximately 32%), followed by mucositis, weight loss, and anaemia. Most toxicities were Common Terminology Criteria for Adverse Events (CTCAE) grade 2. CTCAE grade 3 or 4 toxicities were found in 44 patients (26,8%). All cases of CTCAE grade 4 toxicities consisted of non-febrile neutropenia. Radiotherapy-related toxicity was residual with all but two patients completing the required planned dose.

Assessment of PD-L1 status was included on the pathological report at the moment of diagnosis on a total of 62 patients (38%), while it was not performed (or PD-L1 status was unknown), on the remaining 102 patients (62%). Approximately 35% of patients were PD-L1 positive (which was the majority in the group where PD-L1 was assessed). CPS was defined as <1 (negative), 1-19 and ≥19 (both positive). Approximately 18% of patients had a CPS between 1-19, and 17% had a CPS ≥19.

Disease recurrence was found in approximately 58% (n=95) of patients, with a median time to recurrence of 18 months. Correlation between PD-L1 assessment and main diagnosis, number of treatment cycles, and toxicities were performed through contingency tables and chi-square test for a significance level of 0.05. Relevant results are presented in Table 2. There was no statistically significant relation between PD-L1 value and anatomical location, number of completed cisplatin cycles, or the most frequent CTCAE grade 2 or 3 toxicities of any kind.

**Table 2:** Diagnosis, number of cisplatin cycles, and toxicities according to PD-L1 status SCC: squamous cell carcinoma; CTCAE: Common Terminology Criteria for Adverse Events; PD-L1: programmed death-ligand 1

	PD-L1 positive (n=57)	PD-L1 non-determined or negative (n=107)	Chi-square test χ2	P value
Diagnosis according to anatomical location, n (%)				
SCC oropharynx	19 (33)	41 (38)	6.017	0.305
SCC hypopharynx	17 (30)	24 (22)		
SCC oral cavity	13 (23)	25 (24)		
SCC larynx	7 (12)	14 (13)		
SCC occult primary	1 (2)	3 (3)		
Cisplatin cycles completed, n (%)				
One	18 (32)	25 (23)	1.328	0.515
Three	26 (46)	56 (52)		
Three	13 (23)	26 (24)		
Toxicity grading according to CTCAE, n (%)				
2	47 (64)	30 (69)	0.351	0.553
3	26 (36)	13 (30)		

**Figure 1:**
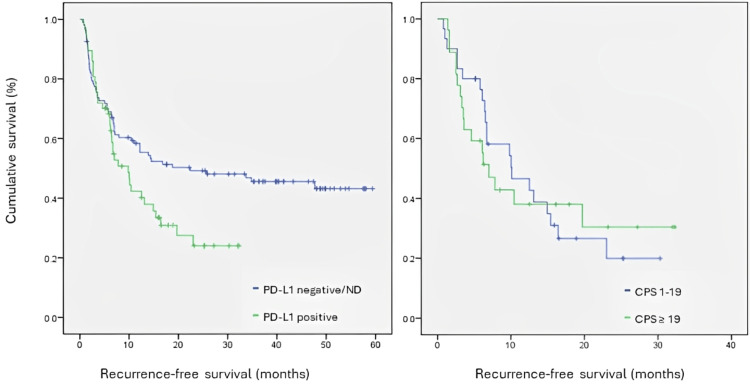
Kaplan-Meyer curve for recurrence-free survival according to PD-L1 status and CPS interval in the PD-L1 positive population PD-L1: programmed death-ligand 1; CPS: combined positive score

**Figure 2:**
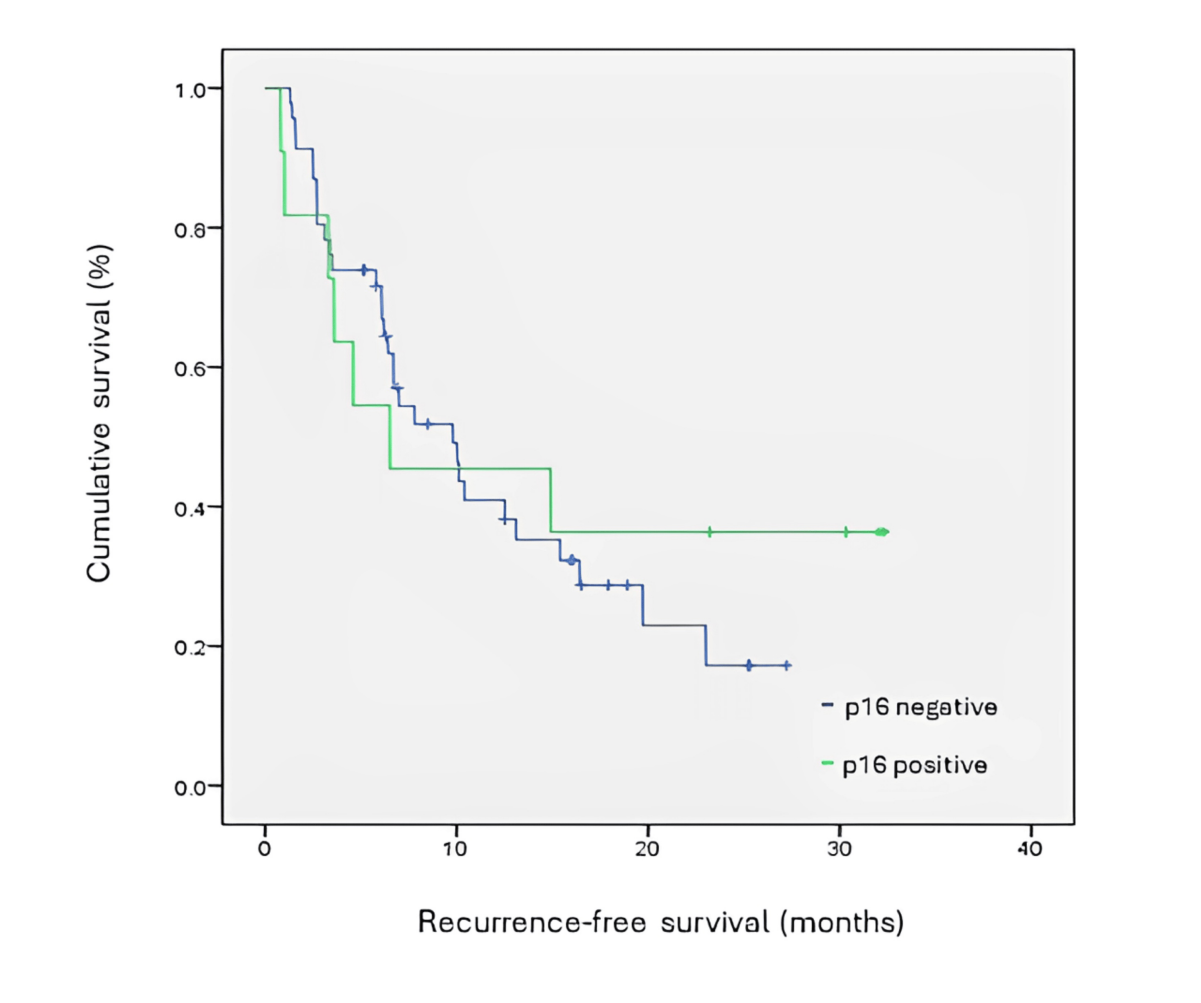
Kaplan-Meyer curve for recurrence-free survival in PD-L1 positive patients according to p16 status PD-L1: programmed death-ligand 1

**Figure 3:**
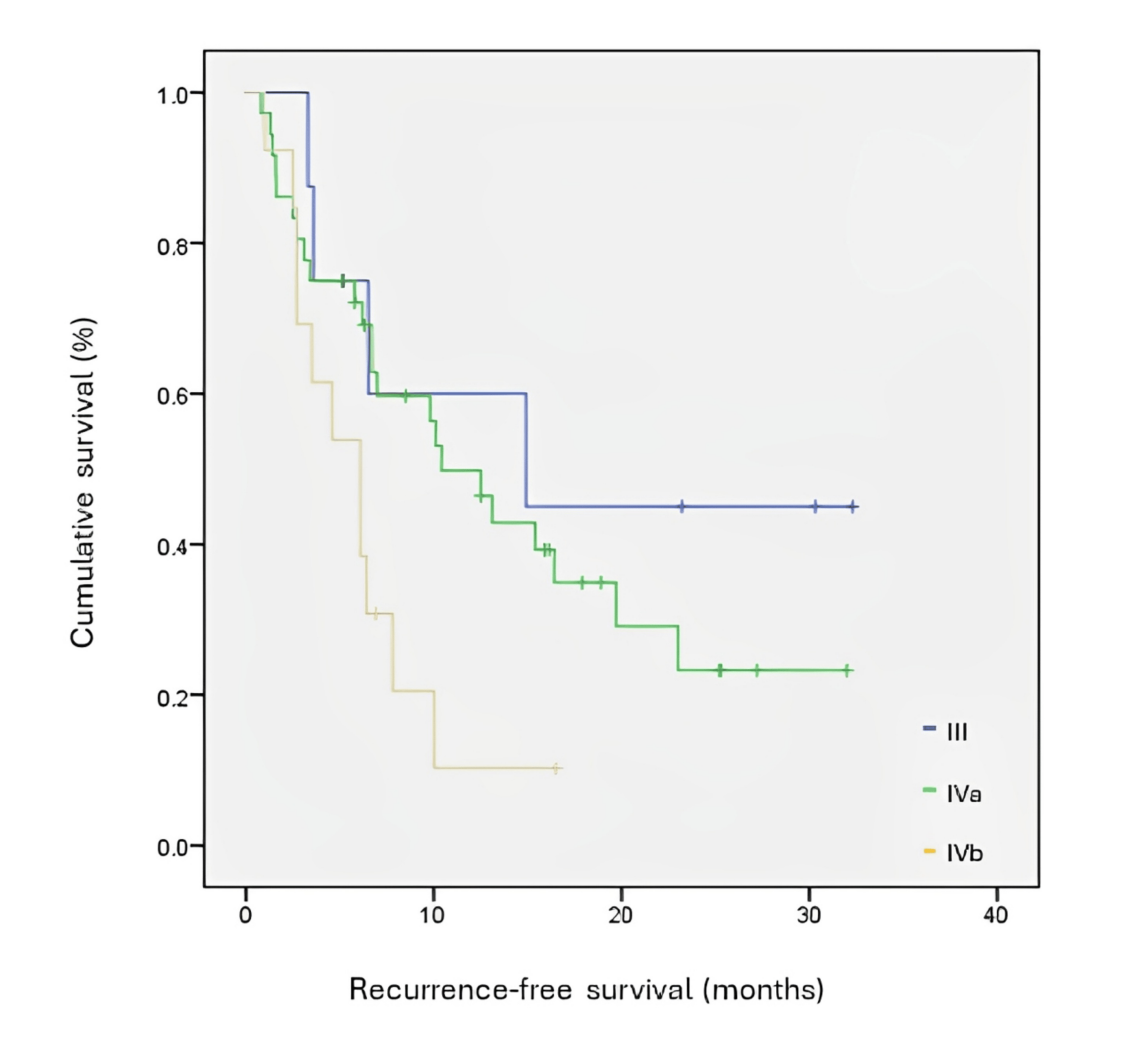
Kaplan-Meyer curve for recurrence-free survival in PD-L1 positive patients according to AJCC clinical staging PD-L1: programmed death-ligand 1

RFS was studied through Kaplan-Meier curves for a level of significance of 0.05. RFS of PD-L1 positive patients was statistically inferior to those in which PD-L1 was negative or non-determined (χ2=4.652; p<0.05). Median time to recurrence was 22 months in the PD-L1 negative/non-determined group and 9,8 months in the PD-L1 positive group. At 20 months, 60% of patients were recurrence-free in the PD-L1 negative/non-determined group, and only 30% were recurrence-free in the PD-L1 positive group. RFS was analysed considering the CPS interval. Although there is a trend for shorter median RFS in patients with CPS ≥19 (7 months versus 10 months in the CPS 1-19 group), this difference was not statistically significant (χ2=0.011; p = 0.916). These results are presented in Table 3 and Figure 1. RFS was analysed between those patients who were p16 positive and negative, in the group where PD-L1 was positive. Statistical significance was not found (χ2=0.180, p>0.671), with a trend for a numerical increase in RFS in those patients who were p16 negative (median time to recurrence 9.8 versus 6.5 months). These results are presented in Figure 2. In those patients who were found to be PD-L1 positive, RFS was analysed according to clinical staging. There was an expected, statistically significant correlation between staging and RFS in this group (χ2=6.409; p=0.041, with a median RFS of 14.9 months for stage III and as low as 6,1 months for stage IVb (Figure 3).

**Table 3:** Recurrence-free survival according to PD-L1 status and CPS interval in the PD-L1 positive population PD-L1: programmed death-ligand 1; CPS: combined positive score

Group	Frequency	Recurrence (n)	Median time to recurrence (months)	Test Logrank χ2	p* value*
PD-L1 positive	57	38	9,8	4.652	0.031
PD-L1 negative/non-determined	107	57	22,4		
CPS 1-19	30	21	10	0.011	0.916
CPS > 19	27	17	7		

## Discussion

Results from trials investigating immunotherapy in PD-L1 positive recurrent/metastatic HNSCC demonstrated statistically significant overall survival benefit, leading to changes in standard of care treatment which now includes immune checkpoint inhibitors, either alone or in combination with chemotherapy. PD-L1 testing by IHC staining is therefore mandatory in clinical practice in this setting, as it guides optimal treatment selection and impacts survival outcomes [11]. On the other hand, the standard-of-care treatment for LA HNSCC has not changed as results from trials introducing immunotherapy in this setting were negative [8-10]. In these trials, PD-L1 was also accessed to guide treatment selection. As such, the determination of PD-L1 in LA HNSCC is not recommended in current guidelines, as it serves no clear clinical purpose.

In some institutions, however, including ours, PD-L1 status may be assessed in LA HNSCC, mostly in order to save time if and when disease recurs, which happens in a significant number of patients. In fact, in our retrospective cohort, an important percentage (58%) of patients had disease recurrence, which was higher than the median rates described in the literature (approximately 40% at five years) [4]. We noted that PD-L1 determination in pathological reports for LA HNSCC has been increasing since 2023, reflecting its importance in clinical trials in this setting.

When present in our cohort, PD-L1 positivity did not seem to correlate to anatomical diagnosis, number of chemotherapy cycles completed or appearance of significant CTCAE grade 2 or 3 toxicities of any kind and, therefore, its determination does not seem to be crucial to predict differences in tolerance to treatment.

Most patients in our cohort were considered locally advanced stage IVa or IVb at diagnosis. Clinical stage IV has been associated with an expected overall increased risk of recurrence [12]. Considering the PD-L1 positive population, RFS was, according to expected, lower in higher stages.

Positivity for p16 in HNSCC, particularly in oropharyngeal cancer, has been associated with longer overall and disease-free survival [13]. The correlation between positivity for p16 and over-expression of PD-L1 is a matter of debate: while some studies have shown a positive correlation to PD-L1 expression [14], others found both expressions to be independent [15]. In our cohort, p16 positivity did not correlate to PD-L1 over-expression in terms of RFS. As such, PD-L1 status also does not seem to be relevant in predicting any unexpected outcome according to clinical staging or p16 status at diagnosis.

Our primary endpoint was assessing RFS in patients with PD-L1 positivity when compared to the general population. Our analysis found that RFS was significantly higher in those patients in which PD-L1 was not determined or negative compared to those in which it was positive. As such, in our series, PD-L1 positivity may represent a group of patients in which treatment failure was higher, recurrence was more common, and prognosis was worse. On the other hand, analysis of RFS taking into account CPS interval analysis found no statistical significance and as such, CPS determination does not seem to play any predictive role. Although studies of immunotherapy in this setting were negative, this real-world data may indicate that further clinical trials are needed. The main limitation of this study is believed to be the fact that a significant percentage of patients in which PD-L1 was not determined, could in fact be either PD-L1 positive or negative.

## Conclusions

Contrary to what occurs in the metastatic/recurrent setting, standard-of-care treatment for LA HNSCC has not changed and there is no current role for immunotherapy in current guidelines. As a consequence, determining PD-L1 expression in this setting of patients is not mandatory. However, PD-L1 status may still be determined if the goal is to save time when and if there is disease recurrence. Our real-world data study aimed to check if there was any other practical role for PD-L1 determination in these patients. While PD-L1 positivity does not seem to correlate to anatomical location, treatment tolerance, p16 status and clinical staging in terms of RFS, it may identify a subgroup of patients in which, globally, recurrence-free survival, and thus, prognosis, is worse. This indicates the need for continued studies on the role of immunotherapy in this setting and justifies discussion on whether PD-L1 determination may play a part in an optimized characterization of these patients.
